# Safety, Feasibility, and Diagnostic Yield of Invasive Coronary Function Testing: Netherlands Registry of Invasive Coronary Vasomotor Function Testing

**DOI:** 10.1001/jamacardio.2024.5670

**Published:** 2025-02-19

**Authors:** Caïa Crooijmans, Tijn P. J. Jansen, Joan G. Meeder, Janneke Woudstra, Martijn Meuwissen, Annemiek M.J. De Vos, Valeria Paradies, Els G. M. Olde Bijvank, Patty Winkler, Nicola S. Vos, Karin Arkenbout, Pier Woudstra, Martin G. Stoel, Tim P. Van de Hoef, Stijn C. H. Van den Oord, Jos W. M. G. Widdershoven, Wouter Remkes, Aysun Cetinyurek-Yavuz, Hester M. Den Ruijter, N. Charlotte Onland-Moret, Eric Boersma, Marcel A.M. Beijk, Yolande Appelman, Jan J. Piek, Regina E. Konst, Angela H. E. M. Maas, Niels Van Royen, Aukelien C. Dimitriu-Leen, Suzette E. Elias-Smale, Peter Damman

**Affiliations:** 1 Department of Cardiology Radboudumc, Nijmegen, the Netherlands; 2 Department of Cardiology VieCuri Medical Center, North Limburg, the Netherlands; 3 Department of Cardiology Amsterdam UMC, Amsterdam, the Netherlands; 4 Department of Cardiology Amphia Hospital, Breda, the Netherlands; 5 Department of Cardiology Catharina Hospital, Eindhoven, the Netherlands; 6 Department of Cardiology Maasstad Hospital, Rotterdam, the Netherlands; 7 Department of Cardiology Haaglanden Medical Center, Den Haag, the Netherlands; 8 Department of Cardiology Maastricht UMC, Maastricht, the Netherlands; 9 Department of Cardiology Onze Lieve Vrouwe Gasthuis, Amsterdam, the Netherlands; 10 Department of Cardiology Tergooi Medical Center, Hilversum, the Netherlands; 11 Department of Cardiology Medical Center Leeuwarden, Leeuwarden, the Netherlands; 12 Department of Cardiology Medical Spectrum Twente, Enschede, the Netherlands; 13 Department of Cardiology UMC Utrecht, Utrecht, the Netherlands; 14 Department of Cardiology Rijnstate Hospital, Arnhem, the Netherlands; 15 Department of Cardiology Elisabeth-Tweesteden Hospital, Tilburg, the Netherlands; 16 Laboratory of Experimental Cardiology UMC/University Utrecht, Utrecht, the Netherlands; 17 Department of Cardiology, Erasmus MC, Rotterdam, the Netherlands; 18 Department of IQ Health Radboudumc Nijmegen, the Netherlands

## Abstract

**Importance:**

Patients with angina and no obstructive coronary artery disease frequently have coronary vasomotor dysfunction as underlying pathophysiological mechanism, comprising epicardial spasm, microvascular spasm, and/or microcirculatory dysfunction. These endotypes can be diagnosed by invasive coronary function testing which has previously shown to be safe in tertiary and expert centers.

**Objective:**

To determine the prevalence of vasomotor dysfunction in patients with angina and no obstructive coronary artery disease who were clinically referred for a coronary function test (CFT); and assess safety and feasibility of a CFT.

**Design, Setting, and Participants:**

This quality improvement study was performed using the Netherlands Registry of Invasive Coronary Vasomotor Function Testing (NL-CFT), a prospective, observational registry, in 15 participating hospitals (2 tertiary and 13 nontertiary). Patients with angina and no obstructive coronary artery disease who were referred for a clinically indicated CFT between December 2020 and January 2024 were included.

**Main Outcomes and Measures:**

A complete CFT consisted of acetylcholine spasm provocation testing and assessment of microcirculatory function. Prevalence of different endotypes based on test results and overall safety were assessed.

**Results:**

Among a total of 1207 patients included, 978 (81%) were female; and the mean (SD) age was 60 (10) years. The prevalence of coronary vasomotor dysfunction was very high (78%). There were 11 (0.9%) major and 10 (0.8%) minor complications reported. Of them, 3 major and all minor were definitely related to the coronary function test. No procedural death, myocardial infarction, or stroke was observed. No differences were found in the occurrence of complications between tertiary and nontertiary centers.

**Conclusions and Relevance:**

This study found that a CFT was feasible and safe to perform in both tertiary and nontertiary centers with a high diagnostic yield.

## Key Points

**Question:**

Is a coronary function test (CFT) safe to perform, and what is the diagnostic yield of the test in patients with angina and no obstructive coronary artery disease undergoing clinically indicated CFT?

**Findings:**

In this quality improvement study of 1207 patients who underwent a clinically indicated CFT, the overall complication rate was 1.7% and major complication rate was 0.9%. Furthermore, the diagnostic yield was high: 78% had abnormal CFT results.

**Meaning:**

These results suggest that CFT is safe to perform with high diagnostic yield and should be considered in all patients with angina and no obstructive coronary artery disease.

## Teaser

This quality improvement study estimates the prevalence of vasomotor dysfunction in patients with angina and no obstructive coronary artery disease who were clinically referred for a coronary function test; and investigates the safety and feasibility of this test in the Netherlands Registry of Invasive Coronary Vasomotor Function Testing (NL-CFT).

## Introduction

Patients with angina and no obstructive coronary artery disease (ANOCA) are prevalent and at increased cardiovascular risk.^1,2^ Coronary vasomotor dysfunction is an important underlying cause, encompassing epicardial spasm, microvascular spasm, and/or coronary microvascular dysfunction (CMD).^3,4^ Diagnosing coronary vasomotor dysfunction by invasive coronary function testing (CFT) with subsequent tailored treatment has proven to be beneficial, resulting in class I and IIa recommendations for CFT for refractory angina in most recent guidelines.^5,6^ However, the majority of safety data come from expert centers. Additionally, differences in CFT protocols hampered widespread incorporation.^7^

A national collaboration initiative, the Netherlands Registry of Invasive Coronary Vasomotor Function Testing (NL-CFT) commenced in 2020.^8^ Participating hospitals perform CFT by a standardized protocol after training by experienced operators. We assessed safety, feasibility, and diagnostic yield of CFT in NL-CFT, comparing tertiary and nontertiary centers.

## Methods

This quality improvement study was not subject to the Medical Research Involving Human Subjects Act in the Netherlands, according to local ethics committees. All participants provided informed consent electronically. The design of NL-CFT and used CFT protocol were published previously.^8^ Coronary angiography is followed by spasm provocation testing and microcirculatory function assessment. The preferred target vessel was the left coronary artery. Seattle Angina Questionnaire (SAQ) was completed at baseline. Included patients underwent clinically indicated CFT between December 2020 and January 2024. The current analysis focuses on the findings, safety, and feasibility of CFT. The cohort was divided between tertiary and nontertiary centers, with tertiary defined as having performed more than 100 CFTs prior to the start of NL-CFT and fulfilling a referral function with specialized ANOCA outpatient clinic.

Intracoronary acetylcholine (ACH) was used for spasm provocation, subsequently administering 2, 20, 100, and maximally 200 µg. Epicardial or microvascular spasm were diagnosed in accordance with COVADIS criteria.^9,10^

Resting and hyperemic measurements using intravenous adenosine are performed to assess coronary flow reserve (CFR) and microvascular resistance.^8^ For cutoff values we adhered to latest available consensus document, despite recent evidence suggesting a more appropriate bolus thermodilution cutoff for CFR of 2.5.^11^ Combining both parts of a CFT results in 6 possible endotypes (see eResults in Supplement 1).

### Safety

Safety was assessed by adverse events and complications. Adverse events included atrioventricular conduction disorders, the need for atropine or the occurrence of ACH-induced atrial fibrillation. A cardioversion for non–self limiting episodes was considered a minor complication. Major complications included death, ventricular arrhythmias, hemodynamic instability with need for inotropes, vasopressors, and/or basic life support, coronary dissection, ischemic event (myocardial infarction, stroke), bleeding, and unplanned hospital admission. All complications were verified in local electronic patient files and discussed during a dedicated NL-CFT meeting.

### Statistical Analysis

Continuous data are presented as mean (SD), categorical data as numbers (percentage). Fisher exact test or χ^2^ test were used for comparison. Differences between groups were assessed by univariate linear regression (analysis of variance). A 2-sided *P* <.05 was considered statistically significant. All analyses were performed using SPSS Statistics version 25 (IBM Corp).

## Results

### Baseline Characteristics

Baseline characteristics are shown in Table 1. Among 1207 included patients, 978 (81%) were female; the mean (SD) age was 60 (10) years. There were 537 patients included in tertiary and 670 in nontertiary centers (Table 2). Anginal complaints were described as pain, pressure, or tightness in the chest by 773 of 841 (92%) while 470 of 841 (56%) experienced shortness of breath. The mean (SD) SAQ summary score was 52 (12). A previous acute coronary syndrome was documented in 234 of 1203 included patients (20%), of which in 94 (40%) myocardial infarction with nonobstructive coronary arteries (MINOCA) was the working diagnosis. One or more diagnostic tests were performed in the majority of the studied population, either in a previous clinical pathway for anginal complaints or in the current, leading to CFT. Noninvasive ischemia testing was performed in 745 of 1178 (63%), with positive results in 96 of 710 (14%) (INOCA).

**Table 1.** Baseline Characteristics

Characteristics	Total cohort	Tertiary	Nontertiary	*P* value^a^
Clinical characteristics				
No.	1207	537	670	NA
Sex, No. (%)				
Female	978 (81)	451 (84)	527 (79)	.02
Male	229 (19)	86 (16)	143 (21)	
Age, mean (SD), y	60 (10)	59 (9)	61 (10)	<.001
BMI, mean (SD)	27 (5)	27 (5)	28 (5)	.16
Classical risk factors, No./total No. (%)				
Hypertension	593/1187 (50)	261/534 (49)	321/653 (49)	.50
Dyslipidemia	523/1171 (45)	233/532 (44)	290/639 (45)	.59
Diabetes	131/1192 (11)	58/535 (11)	73/657 (11)	.88
Current smoker	88/1101 (8)	34/508 (7)	54/593 (9)	.14
Family history of premature cardiovascular disease	506/1033 (49)	240/497 (48)	266/536 (50)	.67
Obesity (BMI ≥25)	748/1148 (65)	334/533 (63)	417/615 (67)	.10
Medical history, No./total No. (%)				
Acute coronary syndrome, of which	234/1203 (20)	100/536 (19)	136/667 (20)	
Myocardial infarction with nonobstructive coronary arteries	94/234 (40)	34/100 (34)	60/134 (45)	.53
Spontaneous coronary artery dissection	9/234 (4)	5/100 (5)	4/134 (3)	
Obstructive CAD	131/234 (56)	61/100 (61)	70/134 (52)	
Percutaneous coronary intervention	252/1204 (21)	107/536 (20)	145/668 (22)	.46
Coronary artery bypass grafting	21/1202 (2)	8/536 (2)	13/666 (2)	.55
Peripheral artery disease	108/1176 (9)	47/533 (9)	61/643 (10)	.69
Migraine	188/823 (23)	126/372 (34)	62/451 (14)	<.001
Autoimmune disease	126/1202 (11)	62/536 (12)	64/666 (10)	.27
Asthma	148/1202 (12)	68/536 (13)	80/666 (12)	.72
Previous diagnostic tests, No./total No. (%)				
Coronary computed tomography^b^	489/1202 (41)	196/535 (37)	293/667 (45)	.006
Coronary angiogram^b^	852/1203 (71)	390/536 (73)	462/667 (69)	.19
Noninvasive ischemia detection test^c^	745/1178 (63)	329/534 (62)	416/644 (65)	
Positive result	96/710 (14)	63/321 (20)	33/389 (9)	.29
Inconclusive result	100/710 (14)	27/321 (8)	73/389 (19)	
Negative result	514/710 (72)	231/321 (72)	283/389 (73)	
Anti-anginal medication use				
β-Blockers	328/1075 (31)	126/469 (27)	202/606 (33)	.02
Calcium channel blockers	705/1075 (66)	307/469 (66)	398/606 (66)	.94
Long-acting nitrates	397/1075 (37)	171/469 (37)	226/606 (37)	.78
Nicorandil	174/1075 (16)	64/469 (14)	110/606 (18)	.05
Ranolazine	2/1075 (0.2)	0/469 (0)	2/606 (0.3)	.51
Angiotensin-converting enzyme inhibitors	173/1058 (16)	86/467 (18)	87/591 (15)	.11
Angiotensin receptor blockers	220/1058 (21)	94/467 (20)	126/591 (21)	.64
Statins	535/1058 (51)	207/467 (44)	328/591 (56)	<.001
Presenting anginal complaints				
Angina, chest pain	773/841 (92)	338/357 (95)	435/484 (90)	.02
Dyspnea, shortness of breath	470/841 (56)	230/357 (64)	240/484 (50)	<.001
SAQ scores, mean (SD)^d^				
No.	601	228	373	
Physical limitation scale	58 (21)	57 (21)	59 (21)	.31
Angina stability scale	47 (24)	48 (24)	46 (24)	.32
Angina frequency scale	59 (22)	57 (23)	60 (22)	.09
Treatment satisfaction scale	70 (20)	69 (18)	70 (21)	.61
Quality-of-life scale	38 (18)	38 (17)	37 (18)	.43
SAQ summary score	52 (16)	51 (16)	52 (16)	.35

Abbreviations: BMI, body mass index (calculated as weight in kilograms divided by height in meters squared); CAD, coronary artery disease; SAQ, Seattle Angina Questionnaire.

^a^ *P* value for comparison between tertiary vs nontertiary centers.

^b^ Previous diagnostic test performed at any point in medical history of patient, not necessarily in current clinical pathway leading to coronary function test referral.

^c^ Exercise test, myocardial perfusion imaging with SPECT, cardiac positron emission tomography, cardiac stress magnetic resonance imaging, or stress echocardiography, included only if not leading to revascularization in past medical history.

^d^ Only included if completed before or within 6 weeks after coronary function test.

**Table 2.** Procedural Characteristics and Safety Outcomes

Characteristics and outcomes	Patients, No. (%)			*P* value^a^
	Total cohort	Tertiary	Nontertiary	
**Procedural characteristics**				
Access, total No.	1204	538	666	
Radial access	857 (71)	450 (84)	407 (61)	<.001
Femoral access	346 (29)	88 (16)	258 (39)	<.001
Conversion after radial attempt, No./total No. (%)	57/337 (17)	33/87 (38)	24/250 (10)	<.001
Size of guiding catheter used, total No.	1171	531	640	
5French	115 (10)	71 (13)	44 (7)	<.001
6French	1056 (90)	460 (87)	596 (93)	
Angiography	1155	529	626	
No angiographic evidence of CAD	450 (39)	232 (44)	218 (35	NA
Nonobstructive CAD	654 (57)	275 (52)	379 (60)	NA
Significant CAD^b^	51 (4)	22 (4)	29 (5)	NA
Physiology				
No.	906	416	419	
Resting full-cycle ratio (RFR), mean (SD)	0.93 (0.04)	0.93 (0.04)	0.93 (0.03)	.80
No.	1045	405	640	
Fractional flow reserve (FFR), mean (SD)	0.90 (0.06)	0.90 (0.06)	0.89 (0.06)	<.001
Function test, No.	1207	537	675	
Acetylcholine test performed	1190 (99)	535 (100)	655 (98)	
Epicardial spasm	504 (42)	256 (48)	248 (38)	.006
Microvascular spasm	307 (26)	158 (30)	149 (23)	
Negative or equivocal	379 (32)	121 (23)	258 (39)	
Adenosine test performed	1170 (97)	516 (96)	657 (98)	.13
Bolus thermodilution, No.	1000	404	596	
Bolus thermodilution coronary flow reserve, mean (SD)	3.9 (1.9)	3.4 (1.6)	4.2 (2.0)	<.001
Index of microvascular resistance	20 (11)	19 (10)	20 (12)	.12
Bolus thermodilution CMD^c^	314 (31)	133 (33)	181 (30)	.41
Doppler flow velocity	157	107	50	
Doppler velocity CFR	2.9 (0.8)	2.8 (0.8)	2.9 (0.9)	.50
Hyperemic myocardial velocity resistance HMR	1.6 (0.6)	1.7 (0.7)	1.6 (0.6)	.37
Doppler velocity CMD^d^	54 (35)	39 (38)	15 (30)	.36
Endotype diagnosis, total No.	1185	532	653	
Normal	258 (22)	72 (14)	186 (29)	<.001
CMD	112 (9)	44 (8)	68 (10)	
Epicardial spasm (isolated)	361 (31)	183 (34)	178 (27)	
Epicardial spasm and CMD	147 (12)	75 (14)	72 (11)	
Microvascular spasm (isolated)	198 (17)	105(20)	93 (14)	
Microvascular spasm and CMD	109 (9)	53 (10)	56 (9)	
**Outcomes**				
Acetylcholine adverse events				
No.	1135	531	604	
Atrioventricular conduction disorders	276 (24)	111 (21)	165 (27)	.01
Atropine administration	16 (1.4)	10 (1.9)	6 (1.0)	.18
Atrial fibrillation	43 (4)	19 (4)	24 (4)	.78
Self-limiting, no further action	33 (77)	13 (68)	20 (83)	.15
Major complications	11/1207 (0.9)	7/537 (1.3)	4/670 (0.6)	.27
Ventricular tachycardia >30 s or ventricular fibrillation	2	1	1	
Hemodynamic instability with need for inotropes	2	2	0	
Persisting spasm despite atropine and nitrates	1	1	NA	
Procedure-related myocardial infarction	0	NA	NA	
Procedure-related cerebrovascular accident	0	NA	NA	
Coronary dissection or other vascular complications^e^	3	1	2	
Ventilatory support necessary	1	NA	1	
Bleeding (BARC>2)	0	NA	NA	
Unplanned hospital admission	2	2	NA	
Procedure-related death	0	NA	NA	
Minor complications, No./total No. (%)	10/1207 (0.8)	6/537 (1.1)	4/670 (0.6)	.34
Atrial fibrillation requiring conversion (chemical), No.	5	2	3	
Atrial fibrillation requiring conversion (electrical), No.	5	4	1	

Abbreviations: BARC, Bleeding Academic Research Consortium; CAD, coronary artery disease; CFR, coronary flow reserve; CMD, coronary microvascular dysfunction; FFR, fractional flow reserve; HMR, hyperemic myocardial velocity resistance; IMR, index of microcirculatory resistance; RFR, resting full-cycle ratio.

^a^ *P* value for comparison between tertiary vs nontertiary centers.

^b^ Defined as angiographically severe, FFR ≤0.80 or RFR or instantaneous wave-free ratio ≤0.89.

^c^ Defined as a bolus thermodilution CFR <2 and/or IMR >25.

^d^ Defined as Doppler velocity CFR <2.5 and/or HMR ≤2.5.

^e^ Access site related; dissection brachial artery, spurious aneurysm.

### Feasibility

Among 1207 patients, conclusive result of both CFT parts was available in 1141 (95%) and CFT was only partially completed in 66 (5%). Two-thirds of these patients with incomplete CFT had at least 1 abnormal result (39% epicardial spasm, 17% microvascular spasm, 11% CMD), confirming coronary vasomotor dysfunction despite not yielding a definite endotype.

### Procedural Characteristics and Diagnostic Yield

Procedural characteristics and endotype distributions are shown in Table 2. Importantly, only 258 patients (22%) had normal CFT results (927 [78%] had abnormal CFT results). The predominant endotype was spasm, either isolated or combined with CMD. Isolated CMD was seen in only 9%. Acetylcholine spasm provocation testing confirmed spasm in 68%. The maximal triggering dose was needed to confirm epicardial spasm in 45%.

Microcirculatory function assessment was performed in the left anterior descending in 98%, circumflex in 1.5% and right coronary artery in 0.5%. Based on bolus thermodilution or Doppler flow velocity measurements, CMD was diagnosed in 31% and 35%, respectively.

### Safety

The overall major complication rate was 0.9%, and no fatal complications occurred. All complications are shown in Table 2. Refer to eMethods in Supplement 1 for a detailed description of complications.

With regards to adverse events, atrioventricular conduction disorders were seen in 24% (95% CI, 22%-27%) of patients. Intravenous atropine to stabilize hemodynamics in conduction disorders or to relieve persisting vasospasm was necessary in 1.4% (0.1%-2.7%) of procedures. Self-limiting atrial fibrillation occurred in 4% (43 of 1135).

### Comparing Tertiary and Nontertiary Patient Populations

Patients undergoing CFT in tertiary referral centers were more frequently female, and arterial access site was more often radial. Vasomotor dysfunction was more frequently diagnosed in patients in the tertiary cohort but was highly prevalent in both groups (86% [95% CI, 84%-89%] vs 71% [95% CI, 68%-75%]; *P* < .001). The major complication rate was 1.3% (95% CI, 0.5%-2.7%) in tertiary and 0.6% (95% CI, 0.2%-1.5%) in nontertiary centers (*P* = .22), the minor complication rate 1.1% (95% CI, 0.4%-2.4%) and 0.6% (95% CI, 0.2%-1.5%) (*P* = .34), respectively.

### Subgroup Analyses

Sex-stratified analyses of endotype distribution and safety were performed. Diagnostic yield was equal for both women and men (78% [95% CI, 76%-81%] vs 76% [95% CI, 72%-83%]; *P* = .61). Endotype distribution was different, with males more frequently showing epicardial spasm. No difference in safety was observed.

## Discussion

### ANOCA and CFT Procedure

We report adherence to current guideline recommendations to perform CFT in patients with ANOCA is safe and feasible in both tertiary and nontertiary centers. The minor and major complication rates were very low, 0.8% and 0.9%, respectively. Among patients with ANOCA, coronary vasomotor dysfunction is highly prevalent, shown in 78% abnormal results. The predominant pathophysiological mechanism was vasospasm. While CMD was observed in approximately one-third of all cases, isolated CMD was rare.

Registries such as NL-CFT help to better understand ANOCA care. The relatively low percentage of radial access is potentially related to smaller radial artery diameter in our predominantly female cohort, or the omittance of nitrates. Reflected by SAQ scores, anginal burden is substantial and often there is an extensive history of diagnostic testing for chest pain. Finally, the prevalence of coronary vasomotor dysfunction was high despite previous negative testing, emphasizing the importance of CFT.^4,12^

### CFT Diagnosis and Endotypes

Previous studies have reported on the prevalence of coronary vasomotor dysfunction in patients with ANOCA. A large meta-analysis of 14 427 patients demonstrated a pooled prevalence of CMD of 0.41 (95% CI, 0.36-0.47), epicardial vasospasm of 0.40 (95% CI, 0.34-0.46), and microvascular spasm of 0.24 (95% CI, 0.21-0.28).^13^ However, these data were acquired with a large variability of methods, definitions and thresholds for diagnosis, rendering direct comparison with our data difficult. Furthermore, the majority was collected in tertiary or expert centers.

### Safety

A meta-analysis of acetylcholine provocative testing included 12 585 ANOCA patients.^7^ Overall pooled estimate of major complications incidence (a composite of death, ventricular fibrillation/ventricular tachycardia, myocardial infarction and shock requiring resuscitation) was very low: 0.5% (95% CI, 0.0%-1.3%). However, these studies were all conducted in specialized centers, which are not necessarily representative of nontertiary centers. Furthermore, the analysis was limited to spasm provocation testing.

Different from earlier studies with acetylcholine infusion, we used intracoronary boluses of acetylcholine, which appear to have a lower complication rate. Our safety outcomes are comparable and numerically lower if limited to the aforementioned definition, demonstrating excellent safety of acetylcholine spasm provocation testing expanding these data with routine CMD assessment.

### Incorporating CFT

An important hurdle to overcome regarding the incorporation of CFT in nontertiary centers are safety concerns. We now demonstrate that CFTs can safely be performed with a low complication rate. In NL-CFT, all CFTs are electively planned, which results in minimal disruption of catheterization laboratory logistics. The procedural time of a complete CFT by a trained operator was 35 minutes on average. With accumulating evidence on the beneficial role of CFT, ad hoc performance after angiography might become future practice.

### Limitations

This study had limitations. First, not all variables were complete in all patients; however, CFT variables were, and all potential complications were checked by the coordinating investigators. Second, our population consisted of females predominantly, reflecting a possible referral bias: subgroup analysis of males showed no difference in the rate of pathophysiological findings. Additionally, the percentage of abnormal CFTs might be lower in a more generalized population.

### Future Perspectives

With regards to pathophysiology and outcomes, follow-up data in NL-CFT will be reported on. Regarding diagnostic processes, some centers have started CMD assessment by continuous thermodilution, a highly reproducible and patient friendly method.^14^ Finally, NL-CFT will serve as a base for randomized therapeutic trials such as the EDIT-CMD trial.^15^ Generally, assessment of noninvasive strategies for the purpose of endotyping coronary vasomotor dysfunction seems an important future field of research.

## Conclusions

Performance of a CFT in the Dutch NL-CFT network including tertiary and nontertiary centers is feasible and safe with a very low overall complication rate of 1.7%. There is a high diagnostic yield among patients who undergo clinically indicated CFT, with vasomotor dysfunction in 78%.

## Notes

Supplement 1.
eMethods

eResults

Supplement 2.
Nonauthor Collaborators

Supplement 3.
Data Sharing Statement
