# Supplementary material for: Safety, Feasibility, and Diagnostic Yield of Invasive Coronary Function Testing: Netherlands Registry of Invasive Coronary Vasomotor Function Testing
Source: JAMA Cardiol. 2025 Feb 19;10(4):384–90. doi: 10.1001/jamacardio.2024.5670 (PMC11840684; doi:10.1001/jamacardio.2024.5670)
Supplement: Supplement 1. — eMethods eResults [file jamacardiol-e245670-s001.pdf]

Supplemental Online Content

Crooijmans C, Jansen TJ, Meeder JG, et al; for the NL-CFT. Safety, feasibility, and diagnostic yield of invasive coronary function testing: NL-CFT. *JAMA Cardiol.* Published online February 19, 2025. doi:10.1001/jamacardio.2024.5670

eMethods

eResults

This supplemental material has been provided by the authors to give readers additional information about their work.

© 2025 Crooijmans C et al. *JAMA Cardiology*.

eMethods

eTable 1. Possible endotype diagnosis after complete CFT.

	**No spasm**	**Epicardial spasm**	**Microvascular spasm**
**Normal CFR/IMR or HMR***	Normal CFT	Epicardial spasm	Microvascular spasm
**Abnormal CFR/IMR or HMR****	Microvascular dysfunction (CMD)	Epicardial spasm with CMD	Microvascular spasm with CMD

CFT coronary function test; CFR coronary flow reserve; CMD coronary microvascular dysfunction; HMR hyperemic microvascular resistance; IMR index of microvascular resistance.

* Normal defined as: bolus thermodilution CFR ≥ 2.0 and IMR < 25 or Doppler CFR ≥ 2.0 and HMR < 1.9.

** Abnormal defined as: bolus thermodilution CFR < 2.0 or IMR ≥ 25 or Doppler CFR < 2.0 or HMR ≥1.9.

© 2025 Crooijmans C et al. *JAMA Cardiology*.

eResults

eFigure 1. Number of coronary function tests per center

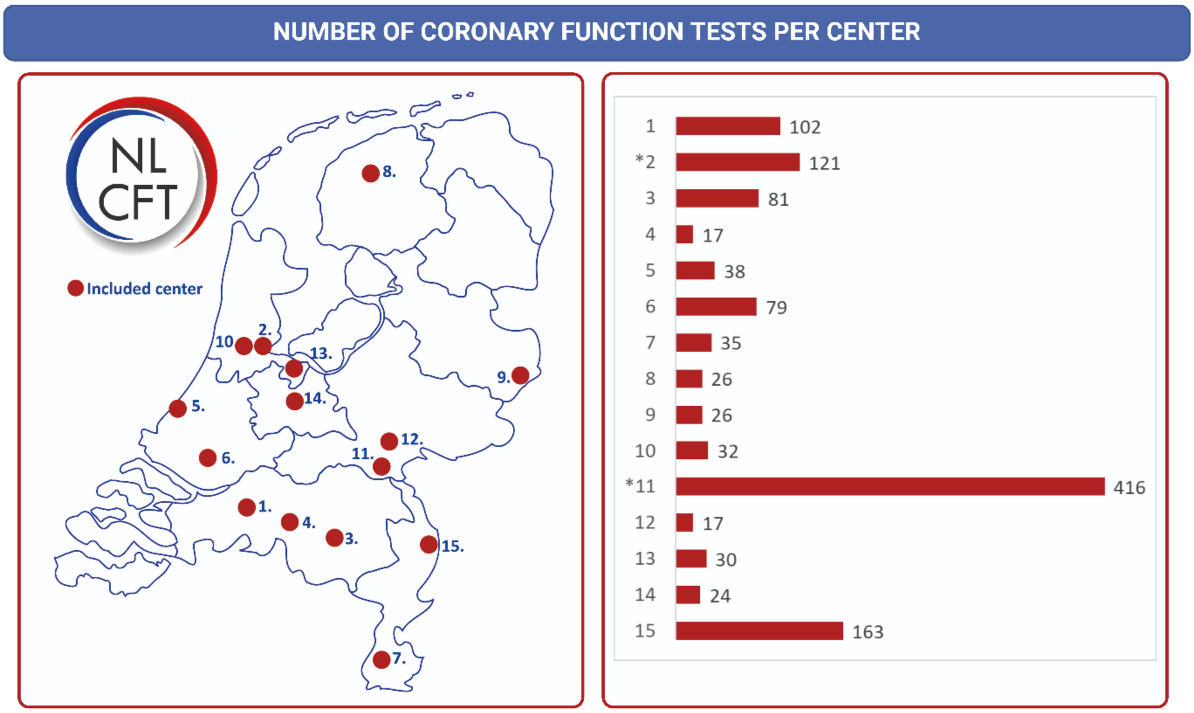

For legend, refer to eTable 2. on the next page.

Created in BioRender. Crooijmans, C. (2024) https://BioRender.com/e63u632

© 2025 Crooijmans C et al. *JAMA Cardiology*.

eTable 2. Participating NL-CFT hospitals

1	Amphia hospital, Breda
2	Amsterdam university medical center, Amsterdam*
3	Catharina hospital, Eindhoven
4	Elisabeth-Tweesteden hospital, Tilburg
5	Haaglanden medical center, Den Haag
6	Maasstad hospital, Rotterdam
7	Maastricht university medical center, Maastricht
8	Medical center Leeuwarden, Leeuwarden
9	Medisch Spectrum Twente, Enschede
10	Onze Lieve Vrouwe Gasthuis, Amsterdam
11	Radboud university medical center, Nijmegen*
12	Rijnstate hospital, Arnhem
13	Tergooi medical center, Hilversum
14	University medical center Utrecht, Utrecht
15	Viecuri medical center, Venlo

Numbers as displayed in Figure 3 of eResults. Hospitals marked with an Asterix were identified as tertiary hospitals.

© 2025 Crooijmans C et al. *JAMA Cardiology*.

Detailed description of all major complications.

Assessment of major complications revealed two cases of ventricular arrythmia. One after intracoronary nitroglycerin administration, presumably due to air bubble injection, solved by defibrillation after which no abnormalities were observed during 3 hours of observation. The other case occurred after angiography of the right coronary artery. A single defibrillation was sufficient to solve the arrhythmia, no prolonged observation was necessary. One case of persisting spasm was seen, nitroglycerin and atropine did not resolve the spasm until 15 minutes later. There was one case of iatrogenic left main dissection by guiding catheter in a patient with a history of transcatheter aortic valve implantation. After placing a stent, patient was admitted for half a day and was subsequently discharged. One patient developed hemodynamic instability following complete left main spasm for which basic life support was provided shortly. After intravenous fluids, atropine and a subsequent admission to cardiac care unit with negative troponin assessments, patient was discharged. Another patient had short loss of consciousness during ACH testing due to 100% spasm of the left anterior descending artery, nitrates and atropine sufficiently resolved the spasm. This event was scored as hemodynamic instability although not requiring inotropes. One patient (without a medical history of asthma) needed temporary ventilatory support with 15 liters of oxygen due to an asthmatic attack after adenosine administration. Two patients developed an allergic reaction for which intravenous medication and prolonged post-procedural observation (6 hours) were sufficient to solve the reaction. These reactions could have been triggered by adenosine, ACH or contrast agent. One patient with right femoral access was treated with a thrombin injection for a spurious aneurysm. He was discharged after uncomplicated CFT and his right femoral was closed with an angioseal closure device. Patient presented himself to emergency department two days after CFT with pain after a forceful movement, when vascular echo revealed a spurious aneurysm, which was successfully treated with a thrombin

© 2025 Crooijmans C et al. *JAMA Cardiology*.

injection. Lastly, there was one case of brachial artery dissection for which the procedure was converted to femoral, no other interventions were necessary.

Analyzing the major complications, two of them were attributable to ACH administration and one to adenosine administration for CMD measurements. The two allergic reactions were potentially related to CFT; they could have been caused by either contrast agent, acetylcholine during spasm provocation or adenosine during CMD assessment.

There were 10 patients (0.8%) in whom acetylcholine triggered new atrial fibrillation for which cardioversion was deemed necessary. These events were counted as minor complications, all attributable to CFT.

© 2025 Crooijmans C et al. *JAMA Cardiology*.
