# Supplementary material for: Safety, Feasibility, and Diagnostic Yield of Invasive Coronary Function Testing: Netherlands Registry of Invasive Coronary Vasomotor Function Testing
Source: JAMA Cardiol. 2025 Feb 19;10(4):384–90. doi: 10.1001/jamacardio.2024.5670 (PMC11840684; doi:10.1001/jamacardio.2024.5670)
Supplement: Supplement 2. — Nonauthor Collaborators [file jamacardiol-e245670-s002.pdf]

Supplemental Online Content: Nonauthor Collaborators
*First name, last name, and suffix (if applicable) are required and will appear in PubMed.

***Group Name(s): NL-CFT**							
***First Name and Middle Initial(s)**	***Last Name**	***Suffix (eg, Jr, III)**	Academic Degrees	Institution	Location (city, state/province, country)	Role or Contribution, eg, chair, principal investigator	Group (if more than 1 Group listed in the byline) and/or Subgroup (eg, Steering Committee)
Wilbert H	Aarnoudse						
Jihane	Bourich						
Bibilotte	Ellerström						
Olaf	Gietman						
Thedde	Kuijsters						
Maribel I	Madera						
Koen MJ	Marques						
Krishan D	Sjauw						
Peter C	Smits						
Bastiaan J	Sorgdrager						
Dirk J	van der Heijden						
Kyra	van Keeken						
Arnoud	van 't Hof						
Tessel N	Vossenberg						
