# Supplementary material for: Safety, Feasibility, and Diagnostic Yield of Invasive Coronary Function Testing: Netherlands Registry of Invasive Coronary Vasomotor Function Testing
Source: JAMA Cardiol. 2025 Feb 19;10(4):384–90. doi: 10.1001/jamacardio.2024.5670 (PMC11840684; doi:10.1001/jamacardio.2024.5670)
Supplement: Supplement 3. — Data Sharing Statement [file jamacardiol-e245670-s003.pdf]

Data Sharing Statement

Crooijmans. Safety, Feasibility, and Diagnostic Yield of Invasive Coronary Function Testing. *JAMA Cardiol*. Published February 19, 2025. doi:10.1001/jamacardio.2024.5670

Data
Data available: Yes
Data types: Other (please specify)
Additional Information: The data underlying this article cannot be shared publicly due to privacy of individuals in the ongoing registry. Where possible, anonymized data queries will be considered and discussed in the NL-CFT steering committee upon reasonable request to the corresponding author. Of note, separate consent was asked to share data with third parties and all requests will need to be examined for eligibility on per patient level.
How to access data: see above
When available: With publication

Supporting Documents
Document types: None

Additional Information
Who can access the data: see above
Types of analyses: see above
Mechanisms of data availability: after approval of a proposal
Any additional restrictions: see above
